# Canine Mammary Carcinomas: A Comparative Analysis of Altered Gene Expression

**DOI:** 10.3390/vetsci3010001

**Published:** 2015-12-25

**Authors:** Farruk M. Lutful Kabir, Carlos E. Alvarez, R. Curtis Bird

**Affiliations:** 1 Auburn University Research Initiative in Cancer (AURIC), Department of Pathobiology, College of Veterinary Medicine, Auburn University, AL 36849, USA; fzl0005@auburn.edu; 2 Current address: Department of Pediatrics, Division of Pulmonology, University of Alabama at Birmingham, Birmingham, AL 35294, USA; 3 Center for Molecular and Human Genetics, The Research Institute at Nationwide Children’s Hospital Departments of Pediatrics and Veterinary Clinical Sciences, The Ohio State University Colleges of Medicine and Veterinary Medicine, Columbus, OH 43205, USA; Carlos.Alvarez@nationwidechildrens.org

**Keywords:** canine, mammary cancer, oncogenes, tumor suppressor genes

## Abstract

Breast cancer represents the second most frequent neoplasm in humans and sexually intact female dogs after lung and skin cancers, respectively. Many similar features in human and dog cancers including, spontaneous development, clinical presentation, tumor heterogeneity, disease progression and response to conventional therapies have supported development of this comparative model as an alternative to mice. The highly conserved similarities between canine and human genomes are also key to this comparative analysis, especially when compared to the murine genome. Studies with canine mammary tumor (CMT) models have shown a strong genetic correlation with their human counterparts, particularly in terms of altered expression profiles of cell cycle regulatory genes, tumor suppressor and oncogenes and also a large group of non-coding RNAs or microRNAs (miRNAs). Because CMTs are considered predictive intermediate models for human breast cancer, similarities in genetic alterations and cancer predisposition between humans and dogs have raised further interest. Many cancer-associated genetic defects critical to mammary tumor development and oncogenic determinants of metastasis have been reported and appear to be similar in both species. Comparative analysis of deregulated gene sets or cancer signaling pathways has shown that a significant proportion of orthologous genes are comparably up- or down-regulated in both human and dog breast tumors. Particularly, a group of cell cycle regulators called cyclin-dependent kinase inhibitors (CKIs) acting as potent tumor suppressors are frequently defective in CMTs. Interestingly, comparative analysis of coding sequences has also shown that these genes are highly conserved in mammals in terms of their evolutionary divergence from a common ancestor. Moreover, co-deletion and/or homozygous loss of the INK4A/ARF/INK4B (CDKN2A/B) locus, encoding three members of the CKI tumor suppressor gene families (p16/INK4A, p14ARF and p15/INK4B), in many human and dog cancers including mammary carcinomas, suggested their important conserved genetic order and localization in orthologous chromosomal regions. miRNAs, as powerful post-transcriptional regulators of most of the cancer-associated genes, have not been well evaluated to date in animal cancer models. Comprehensive expression profiles of miRNAs in CMTs have revealed their altered regulation showing a strong correlation with those found in human breast cancers. These genetic correlations between human and dog mammary cancers will greatly advance our understanding of regulatory mechanisms involving many critical cancer-associated genes that promote neoplasia and contribute to the promising development of future therapeutics.

## 1. Introduction

In the field of human cancer research, there is an intense interest in development of appropriate model systems for the advancement of future therapeutic inventions. Companion animals such as domesticated dogs (*Canis lupus familiaris*) are considered excellent preclinical models of cancers and other complex human diseases for many reasons, including their easy accessibility and living status in diverse cultures [1]. Since they are treated as pet animals, most of the dog population shares the same environment, risk factors or disease characteristics with the human population [2,3] which provides an added advantage for scientists to investigate cancer etiologies. Additionally, dogs represent a more outbred population than inbred laboratory animals providing a genetic diversity similar to that observed in humans [4].

Canine models address two important issues in cancer research. First, in terms of similarities, dogs spontaneously develop cancers in the context of a natural immune system with a clinical presentation, tumor genetics and heterogeneity, disease progression and response to conventional therapies [5] that better models the complex biology of cancers and their interactions with the immune system in human patients than mouse models. The similarities between the dog and human genomes have also greatly enhanced comparative genomic analysis. With the advent of the high resolution 2.4 billion bp canine genome sequence and the identification of nearly all of its genes as clear orthologs of known human genes [4], the dog has emerged as a valuable comparative and intermediate model for the study of human cancers. The high level of sequence conservation between canine and human genomes are key to this comparative analysis especially since 600 Mb of DNA sequence conserved between dog and human is missing from the murine genome [1]. Secondly, using dogs as animal models may contribute to the development of cancer therapeutics for, not only human and dog, but also other species—a promising theme lately coined as “One Medicine” that campaigns under a unified scientific platform where discoveries in one species can be translated to others to improve health management in all species. Canine tumors with potential relevance for human cancer biology include osteosarcoma, mammary carcinoma, lymphoma, melanoma, lung carcinoma, and soft tissue sarcomas [6].

## 2. Canine Mammary Tumors (CMTs)

Mammary tumors are the most common neoplasm in sexually intact female dogs. The severity of canine mammary tumors (CMT) can be appreciated from a number of studies that reported increased rates of incidence in the dog population globally. Breast cancer represents the second most frequent neoplasm in humans and all dogs after lung and skin cancers, respectively, although many reports indicate that dogs are two to four times more susceptible to mammary cancers than women in certain geographical areas [7,8,9,10]. Nearly 50% of all these neoplasms are diagnosed as malignant and more than 95% of these malignant CMTs are carcinomas [11,12].

Canine mammary carcinomas are biologically heterogeneous neoplasms offering several ways to classify such tumors on the basis of histopathological characteristics or expression of molecular markers [13]. Despite the appearance of histomorphological variations between human and canine breast cancers, due to various prognostic indicators, a number of studies have reported that there are significant similarities regarding molecular marker expression, hormone dependency and cancer phenotypes [11,12,13,14]. It is important to classify breast cancer in order to correlate clinical phenotypes, invasion or grade of progression and to develop prognostic markers. The human classification of breast cancers based on expression profile of luminal epithelial specific genes and hormone receptors including estrogen receptor 1 (ESR1), progesterone receptor (PR) and proto-oncogenes such as epidermal growth factor receptors (EGFR/HER2), have also identified similar molecular subtypes in CMTs, but unlike human subtypes, these are not routinely investigated for CMTs during clinical diagnosis [15,16]. Recently, in more refined studies employing immunohistochemical approaches and based on the characteristic expression patterns of ESR1, PR and EGFR (ERBB1/HER1, ERBB2/HER2, ERBB3 and ERBB4), human-like breast cancer phenotypes for CMTs have been developed and classified as luminal A, luminal B, HER2 positive and triple negative (basal-like) [17,18]. Such standard classification therefore strongly supports canine mammary tumors as valuable intermediate models for human breast cancer that should be well-placed for developing diagnostic and treatment strategies.

Because CMTs are considered predictive models for human breast cancer [6], similarities in genetic alterations and cancer predisposition between humans and dogs have raised interest even further. A large number of studies have demonstrated that CMTs have many similarities in molecular and clinical features with human breast cancer. Many genetic/epigenetic/tumor biology traits that are most frequently associated with mammary cancer have been identified and comparative gene expression analysis has revealed a significant similarity in the canine and human genes associated with mammary tumor development [19]. Although CMTs have not yet been classified based on surface markers, due to an absence of appropriate antibodies identifying human breast cancer subtypes, the expression profile of vital genes involved in cellular proliferation, angiogenesis, apoptosis, cell cycle regulation, DNA damage repair, signal transduction, and survival pathways firmly correlate to those in human breast cancer [19,20]. These studies characterized CMTs, based on genome-wide gene expression changes, comparing to human breast cancer, suggesting that mutations and alterations in the cancer genome may promote deregulation of individual genes in mammary cancers.

Comparative analysis of deregulated gene sets or cancer signaling pathways showed that a significant proportion of orthologous genes are comparably up- and down-regulated in both human and canine breast tumors. Prominent oncogenic pathways and related genes, such as PI3K/AKT, KRAS, MAPK, Wnt, β-catenin, BRCA2, ESR1 and P-cadherin, are commonly up-regulated while representative tumor suppressive pathways, such as p53, p16/INK4A, PTEN and E-cadherin, are down-regulated in human and canine breast cancer [19,21,22,23,24,25]. This chapter will discuss the comparative aspects of cell cycle regulatory genes, particularly the evolutionary descent, structure, genomic localization, biological functions, expression defects and post-transcriptional regulation of the CDKN2/INK4 family of cyclin-dependent kinase inhibitors (CKIs) in canine and human breast cancers.

## 3. Cell Cycle Regulators: A Classic Repertoire of Tumor Suppressors

From simple eukaryotes such as yeast to higher mammals, the cell cycle serves as a fundamental biological process by which cells grow and divide and its regulation is central to cancer promoting mechanisms. Cancer causes are complex involving dysregulation of cellular functions and dysregulation in micro-environmental signaling as well as being rooted in oncogenic mutations [26]. Ultimately, cancers occur due to an alteration in the regulation of cell proliferation. Cell proliferation itself is rooted in the cell cycle which is a highly regulated process governed by complex mechanisms [27]. The uncontrolled cell proliferation in cancer is also associated with a vicious cycle where cells divide through unchecked cell cycle progression with a reduction in sensitivity to signals that normally guide cells to adhere, become quiescent, terminally differentiate or die. This combination of unregulated proliferation and a failure of balancing suppressor activities is hallmark of malignant transformation resulting in neoplasia that can eventually develop the ability to spread and migrate throughout the body through metastasis [28]. One such group of genetic alterations that contribute to cancer development are often termed hypermorphic mutations that largely define oncogenes and result from the mutated versions of normal cellular proto-oncogenes. Oncogenic mutations appear to destroy the integrity and modulated control of cell proliferation first by altering control of the stimulatory pathways that promote cell growth. They may also promote neoplasia by suppressing those pathways normally responsible for modulating and inhibiting proliferation or causing exit from the cell cycle entirely. These “loss of function” mutations occur in tumor suppressor genes that encode proteins that can negatively regulate cell cycle progression but, when mutated, are permissive for cancer development and can promote spontaneous as well as, in some instances, hereditary forms of cancer [29]. Two important examples of such loss-of-function mutations affecting cell cycle regulation are mutations in the retinoblastoma (Rb) and p16/INK4A tumor suppressor genes [30,31]. Loss of function of these tumor suppressor gene products results in liberation of the E2F transcription factors, associated with S phase promotion, that consequently remove control of cell cycle exit during G1 phase resulting in abnormal and continuous cellular proliferation [29].

**Figure 1:**
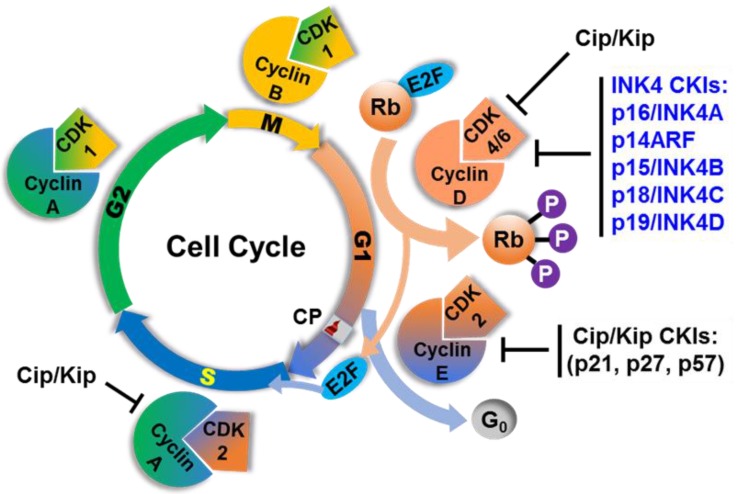
Cyclin-Dependent Kinase Inhibitors (CKIs) are regulators of the cell cycle. Cell cycle phases, major regulatory proteins or protein complexes including cyclins, CDKs, INK4 and Cip/Kip inhibitors and their targets are shown. Checkpoint (CP) or major restriction point.

A group of inhibitory proteins, called cyclin-dependent kinases inhibitors (CKIs) or CDK inhibitors, control cyclin-CDK activity thereby restraining cell cycle progression in response to extracellular and intracellular signals [32,33]. The orderly progression of the cell cycle is fine-tuned by the genes encoding such negative regulators, or CKIs, and positive regulators including the cyclins and CDKs. Dysregulation of these genes can lead to premature and unregulated entry into the next phase of the cell cycle leaving the previous phase unchecked, and frequently this occurs prior to completion of critical molecular events such as repair of DNA damage or replication errors. Such dysfunction frequently triggers genomic instability and neoplastic transformation. Based on their structural similarities and specific roles in cell cycle regulation, CKIs are divided into two distinct groups: the INK4, or CDKN2, and the Cip/Kip, or CDKN1, families [33]. The first group representing the INK4 proteins (Inhibitors of CDK4) are so named because of their ability to specifically inhibit the catalytic subunits of CDK4 and CDK6. It has been reported that INK4 proteins compete with cyclin D for binding to the CDK4/6 subunit [34,35]. The members of the INK4 protein family that share common structural features are p16/INK4A (and p14ARF, an alternatively spliced product from the same locus), p15/INK4B, p18/INK4C and p19/INK4D (Figure 1) [36,37,38]. The Cip/Kip family (for CDK interacting protein/ Kinase inhibitory protein) consists of three members, including p21/Cip1, p27/Kip1 and p57/Kip2, all of which share a common inhibitory domain that enables them to bind CDK complexes [37,39]. These proteins of the Cip/Kip family have broad specificity for binding and inhibiting a number of cyclin-CDK complexes compared to that of INK4 members. They also inhibit the activity cyclin D-CDK4 preventing Rb phosphorylation during G1 to S phase transition. In addition, they inhibit cyclin A-CDK2 in late G1 phase and cyclin E-CDK2 in early S phase (Figure 1) [37]. Therefore, both CKI families are important modulating components of the complex network of cell cycle regulatory mechanisms.

### 3.1. CDK Inhibitors Form a Repertoire of Tumor Suppressor Proteins

Many studies stress the fact that CDKs are positive regulators and CKIs are negative regulators of cell proliferation based on their distinct inhibitory actions in the eukaryotic cell cycle [38,40]. Besides their specific roles in cell cycle regulation, differentiation and development, CKIs are proven or highly likely tumor suppressors to have this potential, as mutations in these genes promote malignant phenotypes [32,36,37,41]. In some clinical trials, CKI tumor suppressors aggressively promote cancer cell growth by inducing p53 function and stability and increasing anti-proliferative activity thereby inhibiting cell cycle progression [42]. Among all the CKIs, p16/INK4A is the founding member and was the first classified as a major tumor suppressor gene (only preceded by p53 for many human malignancies) because the mutations in the INK4A/ARF locus, and loss of heterozygosity of the chromosomal region encoding this gene, have been reported in a wide range of cancers including melanomas, leukemias, gliomas, lung, breast and bladder cancers [36,41]. The loss of expression of the neighboring p15/INK4B gene, due to promoter hypermethylation, also occurs in a number of leukemias and lymphomas [38,43]. The p16/INK4A locus has also been found to be frequently mutated in canine malignant melanomas, mammary carcinomas and fibrosarcomas [21,44,45,46,47].

### 3.2. Evolutionary History, Genomic Localization and Structure of the INK4A/ARF Locus

A locus on the short arm of human chromosome 9p21 (a known multiple tumor suppressor locus) encodes three products called p16/INK4A, p14ARF and p15/INK4B, all of which regulate cell proliferation by inhibiting the cyclin-CDK complex at the G1 to S phase transition in the cell cycle [36,41]. The organization of the INK4A/ARF/INK4B (or INK4A/ARF) locus in the mammalian genome is highly conserved. Orthologous sequence searches and comparative genomics analysis has demonstrated that this locus in human (chromosome 9) is syntenic to that of chimp (chromosome 9), dog (chromosome 11), cat (chromosome D4), mouse (chromosome 4) and rat (chromosome 5) and this region, encoding several tumor suppressor genes, is highly susceptible to genetic instability and mutations in many cancers [36,37,48]. The close similarities between the INK4A and INK4B genes and two other members of the INK4 CKI gene family, INK4C and INK4D based on their protein sequence, biochemical properties and functions in the cell cycle, suggest that they arose as a result of gene duplication during the course of evolution. This is most likely true since a number of studies have demonstrated that all four INK4 CKIs share a common structural feature called ankyrin repeats that appear to function as a structural scaffold facilitating protein–protein interactions and these four CKIs also appear functionally related [36,49,50].

**Figure 2:**
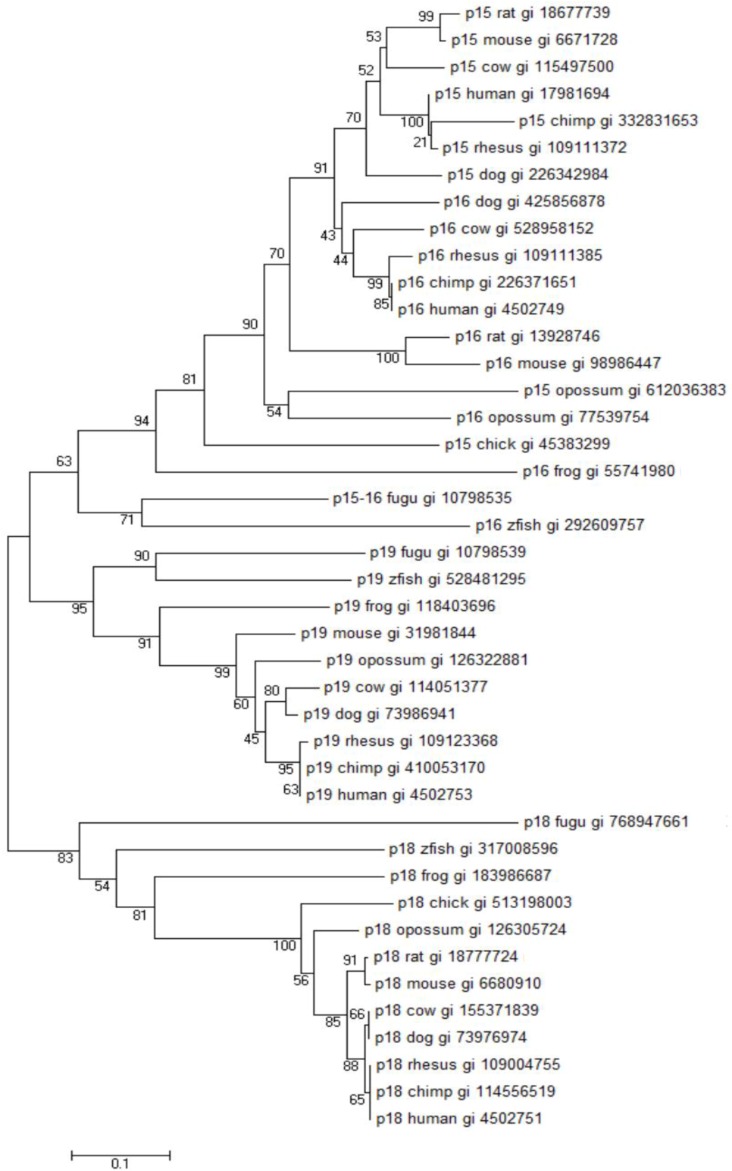
INK4/CDKN2 family tree. Annotated INK4 proteins from select organisms were aligned using Clustal W. The alignment was used to construct a neighbor-joining phylogenetic tree (applying complete deletion of gaps and Poisson model rates and patterns; MEGA6). Bootstrap values were calculated from 500 repetitions. Similar results were achieved with maximum parsimony phylogenetic treeing (not shown). The phylogenetic analyses (see text) demonstrate the high similarity and conservation among INK4 proteins as well as their evolutionary descent. The scale bar shows the number of substitutions per site. NCBI GI accession numbers of the proteins are given on the tree along with the common or abbreviated animal name. Taxonomy abbreviations follow: zfish, *Danio rerio* (zebrafish); fugu, *Takifugu rubripes* (Japanese puffer fish); *Xenopus tropicalis* (western clawed frog); chick, *Gallus gallus* (chicken, red junglefowl); opossum, *Monodelphis domestica*; cow, *Bos taurus*; dog, *Canis lupus familiaris*; rat, *Rattus norvegicus* (Norway rat); mouse, *Mus musculus* (house mouse); rhesus, *Macaca mulatta* (Rhesus macaque); chimp, *Pan troglodytes* (common chimpanzee); human, *Homo sapiens*.

The evolutionary history of the INK4A/ARF/INK4B locus suggests that the INK4 genes have evolved through tandem gene duplication events. One of the most interesting findings for the evolutionary descent of INK4 genes was the complete absence of ARF-like gene products in the Japanese puffer fish *Fugu rubripes* (fugu) and in the zebrafish [37,48] suggesting that p14ARF was introduced into the vertebrate or mammalian genome following INK4 duplication. Three unique INK4 genes, representing INK4A or B, INK4C and INKD have been identified in the fugu genome (Figure 2). Evolutionarily, p16/INK4A and p15/INK4B are products of a local tandem duplication while p18/INK4C and p19INK4D are present on other chromosomes [37,48]. Cross-species comparative analysis suggested that a single common ancestral INK4 gene was present and a series of duplication and rearrangement events first gave rise to INK4A/B and INK4C/D-like elements in a common vertebrate ancestor and after the divergence of higher vertebrates from tetrapod and fish approximately 350 million years ago (MYA) gave rise to the individual INK4 genes in the mammalian genome [37].

These evolutionary changes placed p16/INK4A and p15/INK4B about 30 kb apart in the same transcriptional orientation on chromosome 9p21 whereas p18/INK4C and p19/INK4D are present on human chromosomes 1p32 and 19p13, respectively [36]. Phylogenetic trees based on the published amino acid sequences of INK4 proteins indicates their high similarities among groups and likely divergence from a common ancestor (Figure 2). This evolutionary relationship suggests that the p16/INK4A and p15/INK4B from mammals represent a paralogous group that was once related to p16/15 in fugu or zebrafish while p18/INKC and p19/INK4D are more closely related to corresponding orthologs in fugu. Figure 2 suggests that the complement of INK4 genes arose before the marsupial-placental mammal divergence.

**Figure 3:**
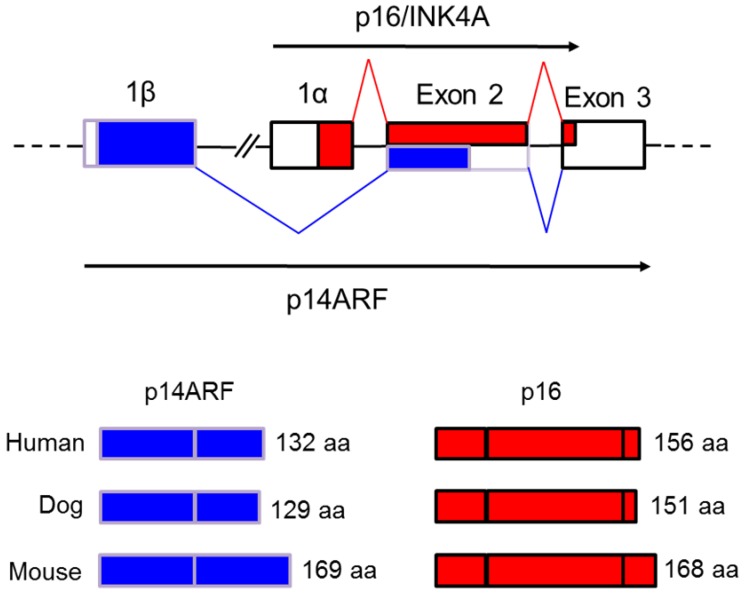
Alternative splicing results in two different transcripts and protein products from the modern INK4A/ARF locus. The exons are shown as boxes and the sequences encoding p16/INK4A are shown as red shading while those encoding the ARF transcript are colored blue. Exon 1α is spliced to INK4A exon 2 and 3 forming the p16 mature transcript whereas exon 1β is alternatively spliced to the same exon 2 and 3 generating the mature p14ARF transcript. The latter produces a different protein from p16 because translation occurs from an alternative reading frame. The sizes of the respective human, dog and mouse p16 and ARF proteins are shown in the bottom panel. (p14ARF in mouse is named p19ARF due to its increased length but should not be compared to p19/INK4D).

During the evolution of p16/INK4A and p15/INK4B through gene duplication, an additional exon appeared when comparing the two genes. This alternative exon is designated exon 1β which is alternatively spliced to exon 2 and 3 of p16INK4A making the novel p14ARF transcript (Figure 3) [37]. Previously, it was postulated that exon 1β was the original exon 1 of the INK4A locus but later it was determined that this alternative exon was transcribed from its own separate promoter and not from the promoter of p16/INK4A exon 1α [51]. The presence of such a separate promoter for p14ARF suggests that its transcription is regulated independently of p16/INK4A. Gene duplication, rearrangement and deletion appear to have resulted in a duplicated exon 1β located in the intergenic region between the INK4A and B genes that later diverged from each other [37,48]. Interestingly, the highly cancer resistant naked mole rat has recently been shown to have an unusual fusion of the p15/p16 tumor suppressor genes (PMID: 25550505). This may represent a further gene rearrangement whose relationship to relative cancer resistance and unusually long life in this rodent remain to be investigated.

### 3.3. Roles of INK4A/ARF Encoded Regulators in the Cell Cycle and Cancer

The existence of p16/INK4A protein was first discovered as a binding partner of cyclin D-dependent CDK4 by the co-immunoprecipitation assay. In cells transformed by SV40 virus, CDK4 was found to be predominantly associated with p16 rather than cyclin D unraveling an important function of this founder member of the INK4 family and suggesting that p16 can directly bind to the catalytic CDK4 subunit in the absence of regulatory cyclin D [40]. Other INK4 members (p15, p18 and p19) were found to interact with CDK4 and CDK6 by two-hybrid screening. Both *in vitro* and *in vivo* studies have reported that all of the four INK4 proteins directly bind the kinase subunits (CDK4/6) rather than the cyclin subunit (cyclin D) as they act as competitive inhibitors of the cyclins [52]. This specific interaction with CDKs distinguishes the INK4 family from the Cip/Kip family of CKIs [36]. Because there is no sequence similarity between exon 1β of p14ARF and exon 1α of p16 and alternative splicing of exon 1β to the shared exon 2 allows translation to continue from the −1 nucleotide of the open reading frame of p16, p14ARF encodes a completely different protein compared to p16. These two proteins also function in distinct biological pathways. Rb is a critical substrate for cyclin D-dependent kinases [40,53] and its phosphorylation is required to release and activate the E2F transcription factors switching on gene expression involved in the G1 to S phase transition [54]. p16/INK4A and the three other INK4 members prevent Rb phosphorylation by inhibiting CDK4/6 binding with cyclin D [34,35]. This cascade pathway in turns leads to E2F repression that inhibits the transcription of many genes required for exit from G1 and initiation of S phase eventually resulting in growth arrest [37,54].

On the other hand, p14ARF is highly unlikely to act as a direct inhibitor of CDK4/6 because of its structural differences from other INK4 proteins. A great number of studies, using mouse models and human cancer cells, differentiated the functions and regulation of p14ARF from that of p16. The initial evidence for its anti-proliferative role came from observations that expression of p19ARF (the p14ARF ortholog in mouse) in embryonic fibroblasts or NIH 3T3 cells induced cell cycle arrest but no direct interaction with CDK complexes was detected in immunoprecipitation assays [55]. It has been reported that loss of p19ARF obviates the requirement of p53 inactivation to immortalize mouse embryonic fibroblasts and tumors, including melanomas, *in vivo* [56,57]. This understanding was further refined by other studies demonstrating that suppression of oncogenic transformation in primary cells by p19ARF is abrogated when p53 is inactivated by viral oncoproteins or dominant p53 mutants [58] implying that p19ARF functions upstream of the p53 pathway. Moreover, some groups reported that p19ARF can associate with MDM2 (a p53 ubiquitin protein ligase) or inhibit the E3-ligase activity of MDM2 to prevent MDM2-induced p53 degradation [57,58,59,60,61] suggesting that these proteins—p19ARF, MDM2 and p53—exist in a common regulatory pathway. In addition to p53 stabilization, p14ARF regulates p53 transactivation activity. p53 normally acts as a strong transcriptional activator of p21/Cip1 protein [38]. Expression of p19ARF in primary mouse cells expressing functional p53 results in the induction of p21 that plays essential roles in G1 to S phase arrest, apoptosis and tumor growth suppression [57,58,59]. Investigating mutations and gene expression profiles of cell cycle regulatory proteins in many human cancer cell lines and primary tumors provided evidence that p53 mutations do not directly correlate with either p16 or Rb expression [30] stressing the fact that p14ARF (in the p53 pathway) and p16 (in the Rb pathway) have distinct or non-overlapping, important biological functions in cell cycle regulation and cancers [36,37]. Thus, this single locus capably regulates the key pathways controlling cell proliferation—the Rb-dependent and p53-dependent pathways.

**Figure 4:**
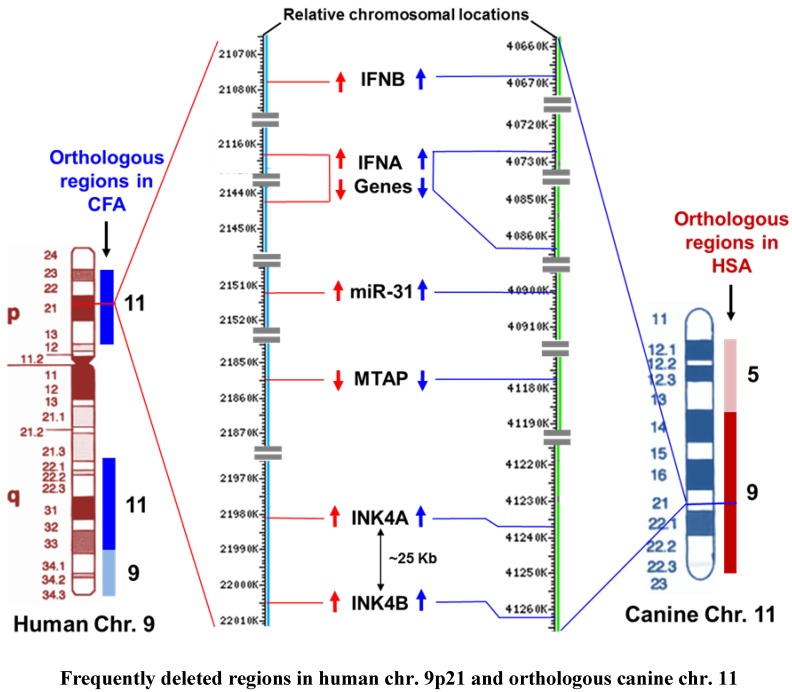
Relative molecular and cytogenetic mapping of the INKA/ARF locus and closely related genes with their positions on human and canine chromosome 9 and 11, respectively. The regions at human chromosome 9 and canine chromosome 11 that are frequently deleted in cancers are completely orthologous to each other. The molecular mapping shows the exact chromosomal position of these genes extrapolated from the NCBI map view of each chromosome represented by the current human and canine annotation from releases 106 and 103, respectively. The red and blue arrows indicate the transcriptional orientation of genes in the human and dog chromosomes, respectively. Transcription of genes from the “+ strand” is indicated by down arrows and from the “– strand” by up arrows. (CFA = Canis lupus familiaris; HSA = Homo sapiens; Chr. = Chromosome).

### 3.4. Alteration of the INK4A/ARF Locus in Human and Canine Cancers

There is compelling genetic evidence from numerous cancer studies that p16/INK4A is a critical tumor suppressor gene whose direct inactivation by point mutation, deletion, or promoter hypermethylation is observed in nearly one third of human cancers, establishing its loss as one of the most frequent lesions promoting human malignancy [37]. The p16/INK4A gene was independently isolated as a candidate tumor suppressor gene located at human chromosome 9p21, the region which is highly conserved across mammals, and was found to be frequently deleted in many human tumors and linked to hereditary susceptibility to melanoma [60,61,62]. The emergence of human chromosome 9p21 as a site of a major tumor suppressor gene was deduced from extensive cytogenetic and loss of heterozygosity (LOH) studies on a wide range of tumors such as leukemias, melanomas, gliomas, pancreatic adenocarcinomas, as well as breast, lung and bladder cancers [61,63,64,65,66,67,68]. LOH of chromosome 9p21 that encodes the INK4/ARF locus was also deleted in the study of a neighboring gene called methylthioadenosine phosphorylase (MTAP) that also mapped to the same chromosomal region [69]. MTAP, a regulatory gene for purine and polyamine biosynthesis, is frequently deleted in different malignant cancer cell lines that also have homozygous deletion of p16 suggesting that loss of MTAP in malignant cells is primarily due to linkage between the MTAP and p16 genes on the same chromosomal region and so they were co-deleted [70]. Furthermore, some malignant cells were found to have homozygous deletion of p16 and MTAP but retained an intact p15 gene. These findings of homozygous deletion of p16 and its neighbor in cancer cells also revealed the gene order on chromosome 9p21 starting from the centromeric end which is p15, (p14ARF) p16, MTAP, IFNA and IFNB (interferon alpha and beta) (Figure 4) [70].

Studies with non-human animal models of cancers have also reported genetic defects in the INK4A/ARF locus. The *in vivo* role of p16 in tumorigenesis was initially indicated from mapping tumor susceptibility alleles in common BALB/c mouse strains. This mouse model is prone to tumor development such as plasmacytoma (tumors of the plasma cells) and lung adenocarcinoma in which the major genetic determinant responsible for a strong cancer predisposition also mapped to the INK4A/ARF locus [71,72]. Mice with targeted deletions of p16, p19ARF or both were investigated by several groups suggesting that mouse strains with specific inactivation of p16 or p19ARF were tumor prone but neither genetic loss alone was as severe as those with double knockouts of both of these genes [57,73,74,75]. Mutant mice that were deficient for p16 and heterozygous for p19ARF spontaneously develop a wide range of tumors including melanoma [95]. Importantly, primary melanomas, mammary carcinomas and osteosarcomas from dogs have also been reported to harbor frequent defects in p16/INK4A [21,45,46,76]. Altered expression profiles from p16/INK4A/ARF have been recurrently observed in a number of canine breast cancers, melanomas and other primary tumors that highly correlate to lesions in humans and mice [30,37,77]. In fact, altogether, deletions or point mutations causing shifting of reading frame and altered expression located mostly in exon 1α have been found in cancers from humans, dogs and mice suggesting that specific mutation mapping in p16/INK4A and its regulation are not limited to these cancer types (for example, melanomas and breast cancers) but also occur in other tumors commonly encountered in mammalian species with neoplasms or uncontrolled cellular growth [21,71,72,77,78].

Furthermore, the region at canine chromosome 11 (orthologous to human chromosome 9p21) encoding INK4A/ARF, MTAP and close neighbors including miR-31, as shown in the comparative chromosomal mapping (Figure 4), is also highly susceptible and prone to concomitant deletion in many cancers in dogs [16]. Reports from several studies suggested that a haplotype spanning MTAP and INK4A/ARF loci showed susceptibility to naturally occurring canine sarcomas [79]. The miR-31, one of the highly cited tumor suppressor miRNAs in human breast cancer, was also found to be down-regulated and differentially expressed in canine osteosarcoma and mammary tumors, respectively [80]. Therefore, the comparative analysis of cytogenetic and molecular mapping of the genetic defects at human chromosome 9p21 and the corresponding canine chromosome 11 identified frequently deleted regions encoded by the INK4A/ARF/INK4B locus with a highly conserved order of genes (Figure 4) that are concurrently lost in many cancers recapitulating the strong similarities in genetic alternations and cancer predisposition between humans and dogs.

## 4. Regulatory, Small Non-Coding RNAs: microRNAs in Cancers

It is increasingly apparent that a significant portion of the mammalian genome (estimated to be >70%) encodes regulatory information that is largely carried out by non-coding RNAs [81,82,83]. These non-coding RNAs consist of two major classes: small non-coding RNAs (<200 bp, including miRNAs) and long noncoding RNAs or lncRNAs (>200 to ~100 kb) [84,85]. lncRNAs share many features of mRNAs, but in contrast to mRNAs, they are found within introns of protein coding genes or intergenic regions of the genome [83] demonstrating developmental and tissue-specific expression patterns [86]. The lncRNAs play a number of important regulatory functions that affect epigenetic changes including chromatin remodeling, transcriptional co-activation and repression, post-transcriptional modification of mRNAs as well as cellular functions including differentiation and homeostasis [84]. Dysregulated expression of lncRNAs causes disruption of these biological functions and plays a critical role in cancer development [85]. To date, a number of lncRNAs have been implicated in breast cancer development and metastasis. One of the most well-known and first identified lncRNAs is a HOX antisense intergenic RNA that is commonly abbreviated as *HOTAIR*. This lncRNA, located in the mammalian HOXC locus, has been demonstrated to be associated with polycomb repressive complex 2 that mediates transcriptional repression of numerous genes involved in differentiation pathways during development and stem cell pluripotency [87,88,89,90]. Importantly, *HOTAIR* has been reported to be highly upregulated in both primary and metastatic breast cancers and its overexpression is a strong predictor of metastasis and poor survival [87]. However, unlike the rapid advances in miRNA research, including the well-established mechanisms of miRNAs in gene silencing and the strong sequence conservation of miRNAs across mammals, knowledge regarding the molecular mechanisms of lncRNA function in cancer is still growing. Most lncRNAs are poorly conserved, and their mechanisms of action remain unclear and are in need of further exploration [83,84].

The discovery of microRNAs (miRNAs) established a new era in translational regulation research and for understanding post-transcriptional regulation of genes as well as their critical regulatory roles in diverse biological processes including cell cycle, cell proliferation, differentiation, development and apoptosis as well as in disease pathogenesis [91,92,93]. miRNAs are evolutionarily conserved, endogenous small structural RNA molecules (~22 nucleotides) that post-transcriptionally suppress gene expression in a sequence specific manner [94]. Expression of these small structural RNAs is tightly regulated during development and in normal mature tissues and is frequently altered in cancer [95]. Strikingly, more than 50% of miRNA genes are located in cancer associated genomic regions or fragile sites that are also preferential sites for translocation, deletion, amplification, and integration of exogenous genome fragments suggesting that miRNAs play an important role in the pathogenesis of many human cancers [96,97]. Since miRNAs are encoded by highly conserved and naturally occurring genes across mammalian species, evaluation of their expression profiles in cancer models shows great promise for advancing the development of future therapeutic reagents, as well as for improving diagnostic and prognostic analysis.

miRNAs and their associated proteins appear to be one of the most abundant biomolecules in the cell. Improvements in small nucleotide amplification technologies and in sequence prediction algorithms, miRNA discovery from model organisms, as well as non-model species, have greatly advanced with 35,828 mature miRNAs in 223 species putatively identified to date (miRBase v20.0). Based on this latest estimate, the human, mouse and canine genomes account for 2588, 1915 and 453 mature miRNAs, respectively, and these numbers reflect those miRNAs identified to date and this is anticipated to grow to similar numbers in most mammals compared to the human genome. The number of experimentally validated miRNAs from each species is smaller than the predicted number. However, both bioinformatics and empirical evidence suggests that more than 30% of protein-coding genes in the human genome are subjected to regulation by miRNAs, indicating their prominence as global regulators of gene expression [98,99,100,101].

In mammals, mature miRNAs generated from sequential processing of primary miRNA transcripts by Drosha and Dicer miRNA processing complexes associate with 3′untranslated regions (3′UTR) of specific target messenger RNAs (mRNAs) to suppress translation and may also induce their degradation [102]. In the nucleus, the RNase III-type enzyme Drosha processes the long primary transcripts (pri-miRNA that is initially transcribed by RNA polymerase II from the cellular genome), yielding 60–70 nucleotide hairpin precursors called pre-miRNA. The resulting pre-miRNA hairpins are translocated to the cytoplasm by Exportin-5. In the cytoplasm, the pre-miRNAs are further cleaved and processed into 19–25 nucleotide miRNA duplex structures by the RNase Dicer and transactivator RNA binding protein (TRBP). The functional strand (or guide strand) of the mature miRNA is loaded together with Argonaut (Ago2) proteins into an RNA-induced silencing complex (RISC), where it guides RISC to silence target mRNAs through mRNA cleavage, translational repression or deadenylation. The passenger strand (the complementary strand of the double stranded pre-miRNA following Dicer processing) is typically degraded [102]. The mature miRNAs usually target the 3′UTR of mRNAs and make complementary base pairing with their seed (core orthologous target) sequences (located at 2–8 bases from the 5′end of the miRNA) [97]. The seed sequence, by which miRNAs bind to their targets, is only several nucleotides long, suggesting that each miRNA may potentially bind to a large number of genes thereby regulating their expression. miRNAs can direct the RISC complex to downregulate target gene expression by either of two post-transcriptional mechanisms: mRNA cleavage or translational repression [98,103,104]. The execution of one of these mechanisms is primarily determined by the degree of complementarity between the miRNA and its target mRNA. The miRNA will promote the cleavage of the target message if its seed region is sufficiently complementary to the target sequences [105]. After degradation of the mRNA, the miRNA remains intact and can guide the RISC to target other messages. Interestingly, miRNAs can regulate their own expression or biosynthesis by targeting the miRNA processing machinery. For example, the miR-103/107 family can inhibit DICER expression and induce epithelial to mesenchymal transition (EMT) promoting metastasis in human breast cancer [106].

### 4.1. OncomiRs: Cancer Associated miRNAs

The association of miRNAs with the initiation, progression and key control pathways of human malignancies holds great potential for new developments in advanced diagnostic and therapeutic strategies in the management of most common cancers. The expression of miRNAs are deregulated in cancer by a variety of mechanisms including amplification, deletion, mutation or epigenetic silencing [107,108,109]. Epigenetic regulation of miRNAs is mediated by promoter hypermethylation in certain human cancers. For example, miR-127, which is downregulated in human cancer cells, has been reported to be located within a CpG island and highly up-regulated by DNA demethylation and histone acetylation [109]. Many groups have discovered “miRNA signatures” in both hematological and solid tumors that discriminate cancers from normal cells and have potential for improving prognosis, management of progression and possibly suppression of cancer [97,110,111,112,113,114]. miRNAs are often regarded as “oncomiRs” meaning miRNAs involved in dominant cancer regulatory mechanisms. OncomiRs can be categorized as tumor oncogenes and tumor suppressors as anti-oncomiRs. miR-155 was one of the first identified oncomiRs that has been demonstrated to be highly expressed in several well-known lymphomas, leukemias, breast, colon and lung cancers [111,113,114,115,116]. Like miR-155, other oncogenic miRNAs usually target tumor suppressor genes and cell cycle inhibitors, or other anti-proliferative genes and they can also serve as potential therapeutic targets. Another strong oncogenic candidate miRNA is miR-21 which is upregulated in a wide variety of blood related and solid tumors including myeloid leukemia, lymphocytic leukemia, gliobalstoma and cancers of the pancreas, prostate, stomach, colon, lung, liver and breast [110,113,117,118,119]. Overexpression of miR-21 in these cancers inhibits the apoptotic pathway promoting dysregulated cell proliferation. miR-21 was also one of the first miRNAs identified in the human genome that showed strong evolutionary conservation across a wide range of vertebrate species. Three major targets of miR-21 include prominent tumor suppressors such as PTEN (phosphatase and tensin homolog), an important regulator of cardiovascular disease, PDCD4 (programmed cell death 4) and TPM1 (tropomyosin 1) [119,120,121,122].

The let-7 miRNA was one of the first anti-oncomiRs, or tumor suppressor miRNAs, characterized, which is highly conserved among mammalian species, and is downregulated in many tumors including lung and breast cancers [111,114,123]. The let-7 miRNA family functionally inhibits a number of well-characterized oncogenes such as *ras*, *c-myc* and *HMGA2* and induces apoptosis and cell cycle arrest in human colon cancer cells [123,124,125,126]. This miRNA targets the *ras* oncogene in lung cancer by being abnormally under-expressed promoting cell cycle progression [123]. In addition, let-7 also downregulates the expression of c-*myc*, a transcriptional activator of many tumor promoting genes that are dysregulated in lymphomas. Thus, anti-oncomiRs effectively control the expression of many oncogenes and their transcription factors at a post-transcriptional level.

### 4.2. Regulation of miRNAs in Human and Canine Breast Cancers

The association between altered miRNA expression signatures and breast cancer metastasis has been described by many studies [127,128]. A large number of miRNAs have been identified as deregulated in human breast cancer compared to normal breast tissue. The overexpression of certain oncogenic miRNAs (miR-21, miR-27a, miR-155, miR-9, miR-10b, miR-373/miR-520c, miR-206, miR-18a/b, miR-221/222) and the loss of several tumor suppressor miRNAs (miR-205/200, miR-125a, miR-125b, miR-126, miR-17-5p, miR-145, miR-200c, let-7, miR-20b, miR-34a, miR-31, miR-30) lead to loss of regulation of vital cellular functions that are involved in breast cancer pathogenesis [127,128]. In human breast cancer, miR-21 upregulates the EMT, the PI3K/ATK signaling pathway, the anti-apoptotic pathway and induces proliferation by targeting very well-characterized tumors suppressors such as PTEN, TPM1, and PDCD4 [121,122,129,130]. Strikingly, all of these miR-21 targets have been reported to be deregulated in canine mammary tumors as well. In this regard, expression of selected miRNAs associated with human breast cancers have been investigated in canine malignant mammary tumors. Almost all of the canine miRNAs in CMTs followed the same expression profile observed in human breast cancers when compared to normal canine mammary tissue. This investigation revealed that miR-21 and miR-29b were significantly up-regulated and miR-15b, miR-16 were significantly down-regulated in breast cancers in both species [131].

### 4.3. miRNAs Regulate Cell Cycle by Targeting Multiple Genes

An important function of miRNAs is to regulate cell cycle progression and arrest by targeting multiple cell cycle regulatory genes. These miRNAs regulate cell proliferation by specifically targeting cyclin-CDK complexes and CDK inhibitors. One of the first discoveries that connected miRNAs and cell cycle regulation was the anti-proliferative potential of the miR-15a/16-1 family that target multiple cell cycle genes involved in cellular proliferation and growth arrest [132,133,134,135]. The miR-16 family act as tumor suppressors that induce cell cycle arrest at the G1 phase by targeting several cyclin-CDK genes including CDK6, cyclin D1, cyclin D3, E2F3 and WEE1 and all the miRNAs in this family are downregulated in a wide variety of tumors [136]. Additionally, miR-34 and other family members, target CDK4/6, cyclin D1, cyclin E2, E2F1/3 and c-myc, indicating their strong anti-proliferative roles [137]. These miRNAs are transcriptionally activated by p53 and are involved in the p53 signaling pathway thereby acting as mediators of tumor growth suppression [138]. However, the tumor suppressive miRNAs involved in cell cycle regulation are inactivated in tumors by epigenetic mechanisms, such as hypermethylation, leading to overexpression of their target genes [139]. For example, members of the miR-290 family positively regulate G1 to S phase transition by inhibiting cyclin-dependent kinase inhibitors such as p21, during embryonic stem cell differentiation [140]. The Cip/Kip family CKIs are targeted by miR-17-92, miR-106b, the miR-221 family and miR-25 in many different carcinomas [136]. Expression of p16/INK4A is repressed by miR-24 and miR-31 which are also involved in the regulation of cell proliferation and progression of cell cycle in many cancers [141,142]. It has been reported that miR-21 negatively regulates cell cycle during G1 to S phase transition in response to DNA damage and inhibits Cdc25A expression affecting G2/M progression in colon cancer cells [143]. Another study showed that miR-322/424 and miR-503 are upregulated during myogenesis and these miRNAs promote cell cycle arrest at G1 phase by down-regulating Cdc25A [144]. A recent report revealed that canine miR-141 can post-transcriptionally regulate p16/INK4A and p14ARF transcripts while groups of differentially expressed miRNAs may potentially target the rest of the CKI gene family members as well as oncogenes of the cell cycle in canine breast cancer models [145]. All of these reports clearly suggest that the cell cycle G1 to S phase transition is tightly regulated by several families of miRNAs. Therefore, different miRNAs regulate the cell cycle both positively and negatively by targeting the expression of many genes at different stages, and dysregulation of most of these regulatory molecules and pathways have been implicated in different pathological or developmental conditions.

## 5. Conclusions

In conclusion, the strong similarities in genome sequence, along with highly similar characteristics for spontaneous tumor models, have raised great promise for further comparative genomic research between humans and dogs. Comparing spontaneous mammary carcinomas in female dogs with breast cancer in women has significantly improved our understanding in deciphering the molecular mechanisms, relevant risk factors, and genetic profiles of these types of cancer and as well as novel strategies for future therapeutic inventions. However, although there is great potential in canine cancer models, a large number and complete interactions of cancer associated genes such as the cell cycle regulators, including the INK4 tumor suppressor genes and emerging miRNAs in the canine genome, have not been well studied in such models. Additionally, the high correlation between tumor suppressor gene expression and miRNA activity imposing post-transcriptional regulation is one of the central areas in cancer research which also needs to be further explored.
